# Comparing the safety and quality of video versus telephone telehealth using simulated consultations on fictional medications for an Australian community sample: a randomised controlled trial protocol

**DOI:** 10.1136/bmjopen-2026-121225

**Published:** 2026-09-30

**Authors:** Yangxueqing Jiang, Yiyun Shou, Eryn J Newman, Bruce K Christensen, Megan Reid Hobbs, Susy Macqueen, Jane Desborough, Danielle Butler, Leanne Watts, Christine Phillips

**Affiliations:** 1 School of Medicine and Psychology, College of Science and Medicine, Australian National University, Canberra, Australian Capital Territory, Australia; 2 Saw Swee Hock School of Public Health, National University of Singapore and National University Health System & Lloyd’s Register Foundation Institute for the Public Understanding of Risk, National University of Singapore, Singapore; 3 School of Psychological Science, University of Western Australia, Perth, Western Australia, Australia; 4 School of Psychology and Social Sciences, The University of Waikato, Hamilton, Waikato, New Zealand; 5 School of Literature, Languages and Linguistics, College of Arts and Social Sciences, Australian National University, Canberra, Australian Capital Territory, Australia; 6 National Centre for Epidemiology and Population Health, College of Law, Governance and Policy, Australian National University, Canberra, Australian Capital Territory, Australia

**Keywords:** Telemedicine, Patient Reported Outcome Measures, General Practice, Cognition

## Abstract

**Introduction:**

Internationally, routine telehealth consultations have been extensively implemented in primary care in telephone or video-based formats. Despite this widespread implementation, few studies have compared information exchange quality in telephone and video-based consultations from consumer and clinician perspectives, nor have they examined related indicators of safety.

This protocol reports on a randomised controlled trial assessing safety and quality of telehealth consultations across telephone versus video-based modalities for simulated consultations about a fictional medication. The primary safety outcome is patient-centred delayed memory recall accuracy as a proxy for medication-information safety. Secondary outcomes include immediate recall accuracy and quality outcomes captured through patient-reported consultation experience and outcome measures. Findings can be used to develop guidance for clinicians and consumers in their selection of the most suitable telehealth modality for medication discussions, ultimately improving primary healthcare delivery.

**Methods and analysis:**

In this study, community participants aged 18–44 complete a simulated telehealth consultation with an actor-general practitioner (GP) through telephone or video, during which they learn about a fictional medication they are advised to take.

Consultation safety will be reflected through participant memory of medication usage items including: frequency, timing, administration instructions, and side effects. Immediate memory will be assessed at the end of each consultation. After a 2-day delay, delayed memory will be assessed using structured recall questions. Consultation quality will be assessed at the end of each consultation through patient-reported outcome measures including: perceived therapeutic alliance with the GP, perceived empathy of the GP, behavioural intentions about medication usage, and risk perceptions regarding medication usage.

**Ethics and dissemination:**

This protocol (ID: H/2024/1249) received ethics approval from the Australian National University Human Research Ethics Committee (HREC) on 14 November 2025. Protocol amendments will be approved by The Australian National University HREC. Study findings will be disseminated through publication in peer-reviewed journals, presentations at academic conferences, and via the study website.

**Trial registration and data statement:**

The trial protocol including a full statistical analysis plan was prospectively registered to OSF (registration ID: osf.io/r6as3) prior to commencement of data collection. De-identified participant data, statistical code, and all materials used will also be uploaded to an online database.

**STRENGTHS AND LIMITATIONS OF THIS STUDY**

- In assessing the safety and quality of telehealth consultations, randomised allocation to modality within a controlled laboratory setting minimises environmental confounds in modality comparison.
- Consultations use piloted fictional medication information matched on recall performance, which supports experimental control but may restrict generalisability to biological-modelled medications with a high-risk side effect profile.
- Findings may not be generalisable beyond the study sample of English-speaking community members aged 18–44, though this demographic represents the highest telehealth utilisation internationally.
- A concern may be that findings reflect actor-general practitioner (GP) characteristics rather than modality differences, which is addressed through equal assignment to modality within actor-GPs, coupled with GP-level statistical analyses.

## Introduction

 After its rapid introduction in 2020 during the COVID pandemic, telehealth consultations have been widely integrated into Australian primary care, representing 20% of general practitioner (GP) services between 2022 and 2023.^1^ During this period, telephone consultations substantially outnumbered video consultations.^1 2^ This preference has persisted despite differential reimbursement under the Medicare Benefits Schedule, where video-based consultations receive higher rebates on the assumption that video consultations carry lower clinical risk and deliver higher quality of care than telephone consultations.^3^ The discrepancy between the policy position favouring video consultations and the apparent preference for telephone consultations in practice indicates an evidence-to-policy gap. There is a need for empirical evidence on modality superiority to support further recommendations regarding modality selection.

Existing literature comparing telephone and video consultations has examined clinical outcomes, cost-effectiveness and perceptions of suitability and safety,^4–7^ with mixed findings regarding modality superiority. While a recent systematic review of telehealth modality comparisons found video consultations to be either superior or equivalent to telephone consultations on clinical outcomes,^5^ two critical quality dimensions remain underexplored: consultation safety and patient-centredness.^8^

In particular, unsafe use of medications has been identified by the WHO as one of the major preventable causes of medical harm.^9^ High quality consultations about medications should involve communication about duration of treatment, purpose of the medication, and the side effect profile of the medication.^10^ Medication safety has been repeatedly identified in systematic reviews as a challenge for telehealth.^11 12^ Video consultations have been identified as resulting in better medication decisions by doctors compared with telephone consultations,^13^ but there are no studies exploring patient reception of medication information across video and telephone.

To-date, one study has compared video, telephone, and face to face consultations using a non-randomised, naturalistic design in general practice.^14^ The study found less information exchange between patients and clinicians in both telephone and video telehealth consultations compared with face-to-face consultations. However, the study was unable to identify differences in information exchange between the two telehealth modalities, and did not examine patient memory across modalities—both key determinants of safety that may be associated with medication non-adherence.^15^ Further, technical factors including connectivity disruption and ambient noise may confound modality comparisons in naturalistic settings.

The current study simulates a clinical telehealth consultation on a recently prescribed, fictional medication in video or telephone format, aiming to assess (1) consultation safety via a proxy of recall accuracy for medication information, and (2) patient-reported quality measures including therapeutic alliance, behavioural intentions, and medication risk perceptions. Specifically, we examine whether these measures are superior in video versus telephone consultations. Findings may be used to inform clinician and consumer decision-making regarding optimal telehealth modality selection, with potential to enhance patient experience and health outcomes.

## Methods

### Patient and public involvement

Consumer representatives were involved in study design by providing input into the authenticity of simulated consultations and supporting recruitment of community members.

### Study design

The current study is a superiority randomised controlled trial to assess whether video telehealth consultation leads to safer consultations and better subjective patient experience than a telephone telehealth consultation when discussing a recently prescribed, fictional medication for sarcoidosis with an actor-GP.

Randomisation is stratified by actor-GP through separate sequence documents for each of the four GPs. For each GP, the lead investigator will generate a random allocation sequence document for 50 numerically-ordered participant IDs, using block randomisation (https://play36.shinyapps.io/Block_Randomization/)^16^ with a maximum block size of 8 where each GP has an even spread of conditions within every 8-person block. Participant allocation to GP will depend on enrolment order and participant-GP availability. After being allocated to a GP, participants will be randomly allocated to (1) a video or telephone telehealth consultation and (2) fictional medication 1 (Kiaroloban) or 2 (Betrixomab), with an allocation ratio of 1:1 for both randomised variables.

### Study participants

#### Population

We target populations aged 18–44 years as the group with the highest utilisation of telehealth in Australia and internationally between 2024 and 2025.^17–19^ This population also controls for age as a known predictor of memory decline.^20^

Participants will be Australian community members who meet the following eligibility criteria: (1) aged 18–44 years at the time of screening, (2) neither themselves nor a first degree relative have sarcoidosis (our hypothetical illness in the case scenarios), (3) be a fluent speaker of English.

#### Recruitment

We will advertise and recruit through Australian consumer organisations, universities, and public spaces using online crowd sourcing and in-person flyers. Participants will receive a $A50 voucher for completing both study phases. Eligibility will be assessed through an online screening survey, after which eligible participants can schedule a consultation. During scheduling, participants will be briefed on the study as involving a simulated telehealth consultation in which they will be discussing a medication recently prescribed for their hypothetical illness (sarcoidosis) and two follow-up surveys.

Potential and enrolled participants’ name, phone number, and email addresses will be collected online and over the phone for consultation booking, recontact, and linking data across phases. This information will be stored securely during the trial and deleted after trial completion.

### Intervention procedure

#### Phase 1

In the initial phase, participants will attend The Australian National University (ANU) School of Medicine and Psychology, ACT, Australia and engage in a simulated telehealth consultation with an actor-GP. Participants will be briefed again on arrival, ensuring the ability to provide informed consent. Research staff will obtain written consent from participants for study participation and use of their de-identified data. Some background briefing about sarcoidosis will be provided.

All consultations will take place in a private laboratory setting with standardised equipment and environment. Participants will be randomly allocated to complete their telehealth consultation on Microsoft Teams via video or telephone. Participants in a video consultation will have their camera and microphone turned on (with self-view hidden) and be able to see and hear the actor-GP. Participants in a telephone consultation will join the Microsoft Teams meeting by dialling in through a provided mobile phone. All consultations will be recorded and transcribed through Microsoft Teams.

##### Simulated consultations

The simulated consultation will be led by a trained actor-GP who will explain how to take the fictional medication by following a standardised consultation script that describes (1) medication frequency, timing, and administration instruction, (2) four side effects for each medication, and (3) interactions with other medications. After each section, the actor-GP will ascertain understanding from the participant and provide clarification where needed using standardised answers that elaborate without providing additional information. The actor-GP can provide, on request, common consultation information irrelevant to each of the key medication items (eg, what to do if a dosage is missed). Actor-GP training aims to maximise GP authenticity while following a standardised delivery style to limit exchanged information to contents of the training manual: the consultation script and answers to common questions.

In each consultation, participants will be randomly allocated to one of two fictional medications (table 1). The medications are modelled on biologics—powerful immune system targeters—which might be initiated for an inflammatory disorder like sarcoidosis. The fictional medications were created by a GP panel to match on risk profile, complexity of use, cognitive-linguistic features, and ease of understanding. Each medication has a set of four side-effects matched on recall likelihood across the two medications (see sample size section for details).

**Table 1:** Fictional medications for sarcoidosis

Fictional medication	Kiaroloban	Betrixomab
Frequency	Once a week	Twice a day
Timing	Take on empty stomach, 1 hour before eating	At morning and night 12 hours apart
Administration instruction	With a glass of water	Swallow it whole
Side effects	Likely to turn urine orange. Increased sweating (1/20 people). Occasionally cause development of a dry mouth. Intrusive suicidal thoughts (1/100k people).	Likely to turn stool dark. Mild acne (1/20 people). Occasionally cause nausea and diarrhoea. Temporary loss of memory (1/100k people).
Interactions with other medications	Aspirin and Nurofen, not alcohol	Aspirin and Nurofen, not alcohol

At the end of the consultation, the actor-GP will employ ‘teach-back’ from the participant to explain (1) how they are going to take the medication and (2) what side effects they need to monitor when doing so. Actor-GPs will frame the purpose of the teach-back as an assessment of whether the GP has communicated information effectively.^21^ Feedback will be provided for both incorrect and correct answers. Errors will be corrected and omissions will be repeated, in line with traditional teach-back methods.^22^ Correct answers will be confirmed through repetition to minimise retrieval-induced forgetting,^23^ as feedback on incorrect answers alone may cause the participant to forget unpracticed, correct answers. Only one round of feedback will be given.

Immediately after the consultation, participants will complete a questionnaire via the computer on perceived therapeutic alliance, perceived empathy, behavioural intentions, and risk perceptions (detailed in Outcome measures). Phase 1 is expected to take 30 min in total.

### Phase 2

To assess delayed memory for consultation content and subjective confidence of memory accuracy, participants will be asked to recall consultation content after a 2-day delay—a common and scientifically established method for testing recall memory.^24 25^ Thus, 48 hours after completing Phase 1, participants will receive an email containing a link to complete an online structured recall questionnaire for Phase 1 consultation content (detailed in Outcome measures).

At the end of the delayed survey, participants will be asked to think back to Phase 1 and indicate whether they experienced any connection issues, and their perception of audio quality and video quality (video quality question only presented to participants allocated to video modality). To ensure data quality, participants will also be asked to indicate whether they wrote down any notes between phases, attempted to recall Phase 1 information as requested, and if they used AI in the study. Phase 2 is expected to take 10 min in total.

### Study management

#### Harms

As the nature of telehealth consultations in this study are simulated, no harms pertaining to discussion of personal and/or health information are expected to arise from completion of the current study. In the event that participants experience concern or distress in Phase 1, actor-GPs and research staff are instructed to follow a distress protocol that includes strategies for checking participation intent, employing grounding techniques, and referral to local support.

#### Retention strategies

Researchers will use email reminders to promote attendance of the consultation during Phase 1 and completion of the delayed online survey during Phase 2.

#### Concealment and blinding

Enrolment staff and coders will be blinded to consultation modality when enrolment/coding is taking place.

Blinding is impracticable for consultation staff, actor-GPs and participants. Consultation staff and actor-GPs will have access to an allocation document, which contains each participant’s first name and the allocated modality and medication for each consultation. Participants are blinded to their consultation modality from enrolment until the consultation. Participants will not be informed of the two intervention conditions until after study completion.

### Sample size and power considerations

Sample size is based on consultation safety as reflected through the primary outcome of delayed recall accuracy, requiring an estimation of baseline accuracy rate. We derive a close estimate of delayed recall accuracy from the recall likelihood of side effects in a pilot study. In the pilot study where participants (n=181) were asked to recall 12 key side effects (8 of which are used in the current study), 74 participants (40.9%) recalled at least three key side effects. As the recall of one out of four possible side effects will be enough for an adequate recall classification in the main study, the lowest consultation safe rate is predicted to be 40%.

The size of the projected difference used in calculations is derived from consideration of the estimated sufficiently important difference (SID).^26 27^ The SID is the smallest clinical benefit to a patient which would justify the associated costs, risks and harms of implementing the more beneficial method. In this case, we have estimated, in conversation with policymakers, that a difference of 25% would be significant enough to warrant the costs and risks of further drivers of uptake of video and reduction in telephone consultation.

We conducted sample size calculations in G*Power using logistic regression with a balanced design of modality. Based on an effect size of OR=2.79 comparing video and telephone consultations on safe rate (65% for video and 40% for telephone; an anticipated clinically meaningful difference), a total of 126 participants provides 80% power to observe the difference at a Type 1 error rate of 0.05 in a two-tailed test. We will continue to recruit participants until valid data from participants who complete both phases reaches the desired sample size (n=126; 63 participants per modality).

### Study timeline

Recruitment began on 27 March 2026. The study is estimated to reach the desired sample size by 31 December 2026. A time point schedule of the full study is shown in table 2.

**Table 2:** Schedule of enrolment, comparator, assessments, and contact

	Trial period					
	Enrolment		Study phases			Closeout
Time point	Prior to 0	0	Phase 1	Post Phase 1	Phase 2	
Enrolment						
Informed consent			x			
Eligibility screen	x					
Randomisation	x					
Consultation slot booking	x					
Comparator						
Video or telephone consultation			x			
Assessments						
Teach-back			x			
End of consultation survey			x			
Delayed survey					x	
Contact (participants)						
Confirmation of consultation slot		Email				
Reminder for Phase 1		Email				
Link to Phase 2 survey				Email		
Reminder for Phase 2 survey (if non-completed within first 12-hour window)				Email		
Remuneration gift card						Email
Email contact (GPs)						
Confirmation of shifts		x				

GPs, general practitioners.

### Outcome measures

#### Primary safety outcome: Delayed recall accuracy

*Delayed memory accuracy*. The primary goal of a telehealth consultation on medication usage and safety is for patients to comprehend and remember information discussed accurately after a delay. The primary outcome measure will be a dichotomous recall classification (adequate/inadequate) for each consultation as a proxy for consultation safety. This classification will be based on recall accuracy as scored by assessing Phase 2 survey answers for key medication usage items: frequency, timing, administration instruction, and side-effects. Minimum requirements for adequate recall are correct answers to frequency, timing, administration instruction, and at least one side-effect, as informed by elements of Action V.4.03 (Partnering with Consumers) of the national Medication Safety Standard.^10^ While side-effects are composed of both the likelihood of occurrence and a key outcome (eg, occasional sunburn), recall accuracy will be based on outcome only, as most pilot study participants were unable to recall likelihood after 48 hours. A global ‘inadequate’ assessment will be made if the person cites the medication dosage (derived from response for frequency) at a higher than prescribed daily dosage or fails to meet safety criteria.

Notably, delayed memory accuracy reflects the effects of modality and standardised reinforcement during teach-back, rather than the effects of modality alone as captured in immediate recall accuracy below. We will code teach-back content and participant questions during the consultation to allow for a closer examination of these factors in exploratory analyses.

*Immediate memory accuracy*. We will use consultation transcripts to assess participants’ memory for key medication usage items in the teach-back session before the end of each consultation. These assessments will be used to classify immediate recall using the same dichotomous adequate/inadequate classification as above.

#### Secondary outcomes: Quality outcomes

We will draw on several quality variables to compare outcomes of telephone versus video telehealth modalities.

*Therapeutic alliance*. A 5-item telehealth patient experience measure will be used to assess therapeutic alliance between the patient and GP^28^—a critical safety target that has been shown as tied to beneficial health behaviours, treatment adherence and patient satisfaction.^29^ These items evaluate perceived effectiveness of the GP’s communication and perceived ability to build connection and trust with the GP, with good internal reliability (Cronbach’s alpha >0.87).^28^ All items will be aggregated into a single therapeutic alliance measure.

*Perceived empathy of clinician*. We will use the Consultation and Relational Empathy (CARE) measure^30^ to evaluate convergent validity with the five-item telehealth patient experience measure of therapeutic alliance. The 10-item CARE measure is a widely used measure of consumer’s view of clinician empathy and consultation quality, validated in primary and secondary care contexts with good internal reliability across care contexts and languages (Cronbach’s alpha >0.90).^30^ Perceived empathy is a key determinant of consumer trust in the clinician and their willingness to engage in video consultations and shared decision-making.^31^ All items will be aggregated into a single CARE measure.

*Behavioural intentions*. As another measure of participants’ trust in the GP, we will use three behavioural intention items regarding medication usage, with demonstrated good internal reliability (Cronbach’s alpha >0.94).^28^ All items will be aggregated into a single behavioural intention measure.

*Risk perceptions*. We will ask participants to subjectively assess the risks involved in taking the medication incorrectly. We follow the theoretical construct of risk perception to capture two major components: susceptibility and severity, using four items with similar wording to existing questions used in studies on health risk perception.^32 33^ We will assess reliability of all four items (one reverse-coded) before aggregation.

#### Exploratory quality outcomes

*GP-rated quality*. GP registrars will review consultation recordings and provide subjective impressions of the consultation on all quality outcomes, using the same scales that were provided to participants (adapted from first to third person). This allows measurement of the extent to which GP impressions in telephone and video consultations align with more objective measures of patient safety (eg, memory performance). We will use stratified random sampling to select a representative subset of consultations using stratification by key variables: consultation modality (telephone/video), participant demographics, and actor-GP cluster.

## Data methods

### Data/conduct monitoring

There will be no data monitoring committee as the study is not a clinical trial. However, a steering committee will perform a function that supersedes the roles of a data monitoring committee.

GP team members will review recordings of the first five consultations held by each actor-GP to provide feedback that aligns consultation delivery manner with the standardised procedure instructed during actor-GP training.

### Data management

Data management procedures are outlined in the data management plan (DMP) (online supplemental file) created using the DMP Tool in accordance with ANU guidelines.

All surveys will be collected separately via Qualtrics and then combined into a single dataset for analysis. All documents, data and recordings will be saved to secure ANU SharePoint sites created for the protocol project, with access restricted to the research team.

As per research ethics and legislative guidelines, all surveys and recordings/transcriptions will be retained for a minimum of 5 years after the completion of the study. Beyond the initial storage period, the anonymous data will remain archived in publicly accessible data repositories, giving access to other researchers as per journal requirements.

De-identified data collection forms will be linked in the repository of the published paper reporting study outcomes.

### Data preparation

#### Exclusions

Participants who do not complete Phase 2 will be excluded from analysis of delayed memory accuracy. If the number of Phase 2 non-completers exceeds 10% of the total sample, we will assess potential bias introduced by differential attrition between conditions by comparing available baseline characteristics (age, end-of-consultation survey completion rate, experimenter-assessed technical difficulty of consultation) between completers and non-completers using χ² tests and independent samples t-tests. We will report the proportion and characteristics of excluded cases. All other randomised participants will be included in data analysis below.

#### Missing data

We will examine patterns of missing covariate data to identify the pattern of missingness. We will use multiple imputation for data missing at random^34^ and multiple imputation by chained equations for data missing not at random.^35^ Scales missing more than 30% items will be excluded from analysis.

Missing recall components are not treated as missing data, as they reflect memory accuracy.

### Data coding

Two independent coders (YJ and MRH, who also scored the pilot study data) will score (1) immediate accuracy of each key medication item as immediately reported by participants in the teach-back phase in response to the teach-back request, before feedback is given by the actor-GP and (2) delayed recall accuracy of each item in the Phase 2 survey. Coders will use item scores to determine immediate and delayed overall safety classification for each consultation.

Coders will also track participant questions during the consultation itself and code, for the item that the question is related to, the relevant question category: informational (seeking extra detail), clarification (seeking understanding), or advice (seeking related advice). These data may be used in exploratory analyses to better understand the role of clarification and information seeking in delayed recall performance.

As in the pilot study, coding disagreements will be resolved by the chief investigator CP as adjudicator. In the pilot study, the appointed coders reached inter-rater agreement of 100% after resolving disagreements and correcting errors.

### Statistical analysis plan

All analyses will be conducted in R. No interim analyses are planned for this study.

#### Primary safety analysis

To examine differences in dichotomous consultation safety outcomes between telephone and video modalities, we will use two simple logistic regression models testing whether consultation modality (telephone vs video) predicts recall adequacy (adequate/inadequate) as determined by (1) immediate recall accuracy or (2) delayed recall accuracy. Results will be reported as ORs with corresponding 95% CIs, with statistical significance set at α=0.05.

Relevant covariates are included to control for confounding. Potential covariates include: medication assignment, age and gender. We will select covariates using a systematic approach, considering variables with p<0.25 in univariate analyses for inclusion in adjusted models.^36^ The final model will balance statistical and clinical considerations while avoiding overparameterisation.

#### Secondary safety analysis

To examine the robustness of the primary safety analysis across actor-GPs, we will use two mixed-effects logistic regression models for safety outcome with modality (telephone vs video) as the fixed effect and actor-GP as a random intercept to account for clustering of consultations within practitioners, again for immediate then delayed memory. Results will be reported as ORs with corresponding 95% CIs, with statistical significance set at α=0.05. We will report the intraclass correlation coefficient to quantify the proportion of variance attributable to GP-level clustering. Model fit will be evaluated using the Akaike information criterion.

#### Primary quality analysis

To compare patient-centred quality outcomes across modalities, we will run a separate linear mixed-effects model for each of the quality outcomes: perceived therapeutic alliance, perceived empathy, behavioural intentions, and risk perceptions.

#### Exploratory quality analysis

We will also perform the above multivariable logistic regression models with GP-rated covariate measures to assess whether GP-rated measures or participant-rated measures are better predictors of consultation safety.

#### Exploratory modality analysis

We may also explore other outcomes between modalities, including but not limited to: recall confidence, audio quality, video quality (only for video modality), participant questions during the consultation, and actor-GP feedback during the consultation.

## Limitations

The current study has several limitations. While actor-GP training was designed following best practice guidelines to minimise GP-based confounds,^37 38^ resources limited the selection of actor-GPs from being balanced on factors that may influence the doctor–patient relationship. Simulation-specific confounding variables have been consistently identified as a threat to internal validity in applied medical research.^37 39^ In the current study, actor-GP-related confounds will be addressed by enabling within actor-GP analysis through consistent modality distribution (1:1). Further, the generalisability of findings is limited to English-speaking populations aged 18–44, consultations with actor-GPs, and fictional medications.

## Discussion

This study aims to examine the safety and quality of telehealth consultations across video and telephone modalities for participants aged 18–44. We will assess consultation safety through proxy safety measures—immediate and delayed memory accuracy—for medication information delivered during the simulated consultation, and consultation quality through patient-centred measures of consultation experience and outcomes.

## Supplementary material

10.1136/bmjopen-2026-121225 online supplemental file 1
