# Supplementary material for: Comparing the safety and quality of video versus telephone telehealth using simulated consultations on fictional medications for an Australian community sample: a randomised controlled trial protocol
Source: BMJ Open. 2026 Sep 30;16(9):e121225. doi: 10.1136/bmjopen-2026-121225 (PMC13629935; doi:10.1136/bmjopen-2026-121225)
Supplement: Supplementary file 1 [file bmjopen-2026-121225.reviewer_comments.pdf]

PEER REVIEW HISTORY

BMJ Open publishes all reviews undertaken for accepted manuscripts. Reviewers are asked to complete a checklist review form and are provided with free text boxes to elaborate on their assessment. These free text comments are reproduced below.

ARTICLE DETAILS

Title (Provisional)

Comparing the safety and quality of video versus telephone telehealth using simulated consultations on fictional medications for an Australian community sample: a randomised controlled trial protocol

Authors

Jiang, Yangxueqing; Shou, Yiyun; Newman, Eryn J; Christensen, Bruce K.; Reid Hobbs, Megan; Macqueen, Susy; Desborough, Jane; Butler, Danielle; Watts, Leanne; Phillips, Christine

---

VERSION 1 - REVIEW

**Reviewer**	**1**
**Name**	**Heidari-Soureshjani, Reza**
**Affiliation**	**Tehran University of Medical Sciences School of Nursing and Midwifery**
**Date**	**22-May-2026**
**COI**	**None**

This is a relevant protocol addressing an important question. Several issues should be clarified before publication..

The manuscript overstates medication safety and clinical implications. The study evaluates recall of fictional medication information and patient experience in simulated consultations. It does not assess real medication use, medication errors, adverse events, adherence, or clinical outcomes. The primary outcome should therefore be described as a recall-based proxy for medication-information safety, not medication safety itself. Please revise this framing throughout the manuscript and temper the conclusions accordingly. The limitations should also acknowledge the restricted generalizability of a laboratory study involving English-speaking community participants aged 18 to 44 years, actor-GPs and fictional medications.

The primary endpoint and safety classification are also unclear. The protocol labels immediate recall, delayed recall, and several patient-reported measures as primary outcomes. Please identify a single primary endpoint and time point, state which endpoint

informed the sample size, and classify the remaining outcomes as secondary or exploratory. The safe/unsafe classification needs justification as well: interactions are assessed but not required for a safe classification, recall of only one side effect is considered sufficient, and the outcomes refer to "dosage" although Table 1 reports frequency and administration instructions rather than an actual dose. Reporting component-level recall alongside the global classification would improve interpretation.

A few aspects of teach-back, randomization, and outcome assessment also need clarification. Because actor-GPs correct incorrect responses and repeat correct responses during teach-back, delayed recall reflects modality plus standardized reinforcement rather than modality alone. Please clarify whether immediate recall is scored before feedback and how corrections, clarification requests, and protocol deviations will be documented . Participants are also randomized to one of two fictional medications that differ in instructions and side-effect content, so medication assignment should be included in the analysis. Allocation concealment is unclear because enrolment staff and actor-GPs appear to have access to allocation documents; please explain how foreknowledge of assignments is prevented. Finally, since the primary outcome is transcript-coded, please describe coder blinding, inter-rater agreement, and adjudication procedures.

The sample size and analysis plan are not fully aligned. The sample size calculation uses simple logistic regression with a one-tailed test, whereas the planned primary analysis uses mixed-effects logistic regression with actor-GP as a random intercept. Please justify this alignment, the assumed effect size, the one-tailed test, and the use of a random effect with only four actor-GPs. Selecting covariates through univariate p values is generally not recommended for a randomized trial primary analysis; any adjusted covariates should be prespecified. The missing-data plan also needs clarification, since excluding phase 2 non-completers may bias delayed-recall comparisons, and MICE alone does not address Missing Not at Random assumptions.

Several protocol reporting details remain incomplete. The patient-reported measures need clearer description, particularly the therapeutic alliance measure cited as a manuscript in preparation, which cannot be independently appraised. Please provide scoring, timing, missing-item handling, and available validity and reliability information for each measure; adapted GP-rated versions should be considered exploratory unless validated. Please also clarify actor-GP training and fidelity monitoring, technical disruptions, and whether feedback after the first five consultations may alter delivery during data collection. Finally, please report recruitment status and study dates, clarify whether the trial was prospectively registered in a registry acceptable under journal policy, and strengthen the harms section by describing distress monitoring and debriefing, particularly given the inclusion of "intrusive suicidal thoughts" in the fictional medication information.

A few smaller points to consider as well. The title would benefit from indicating that the study involves simulated consultations and fictional medication information. The Discussion

reports an age range of 40 to 65 years, whereas the eligibility criteria specify 18 to 44 years. Professor Bruce Christensen appears in the Author Statement but not in the author list, so authorship status should be clarified. Please provide competing-interest disclosures for all investigators and state the funder's role. Terminology should be consistent for telephone/audio consultations and for de-identified/anonymous data. The Fakhrai reference also appears incomplete.

Thank you.

VERSION 1 - AUTHOR RESPONSE

Reviewer 1 comments to Author:

This is a relevant protocol addressing an important question. Several issues should be clarified before publication.

1. *The manuscript overstates medication safety and clinical implications. The study evaluates recall of fictional medication information and patient experience in simulated consultations. It does not assess real medication use, medication errors, adverse events, adherence, or clinical outcomes. The primary outcome should therefore be described as a recall-based proxy for medication-information safety, not medication safety itself. Please revise this framing throughout the manuscript and temper the conclusions accordingly.*

   Response: Thank you for this feedback. We have now addressed this issue and revised the primary outcome framing as a "recall-based proxy for medication-information safety". This framing is employed in the Abstract introduction, protocol introduction, and protocol discussion.

2. *The limitations should also acknowledge the restricted generalizability of a laboratory study involving English-speaking community participants aged 18 to 44 years, actor-GPs and fictional medications.*

   Response: We have addressed these limitations in the "Strengths and Limitations" section: "Further, the generalisability of findings is limited to English-speaking populations aged 18-44, consultations with actor-GPs, and fictional medications." (page 12)

3. *The primary endpoint and safety classification are also unclear. The protocol labels immediate recall, delayed recall, and several patient-reported measures as primary outcomes. Please identify a single primary endpoint and time point, state which endpoint informed the sample size, and classify the remaining outcomes as secondary or exploratory.*

   Response: The primary outcome that informed sample size is delayed recall, which we now emphasize in "Sample Size and Power Considerations" section under Methods that "Sample size is based on consultation safety as reflected through the primary outcome of delayed recall accuracy". (page 7) We hope that our revisions now make it clear that the sample size calculation was based on the recall accuracy, rather than safety classification.

   As the aim of the current protocol is to compare consultation modalities on both safety and quality, we retain the two primary outcomes "Safety Outcome: Medication safety" (coded based on recall accuracy) and "Quality Outcomes", where we note that "We will draw on several quality variables to compare outcomes of telephone vs video telehealth modalities." (page 9)

   The final phase (end point) of the study is where people complete the delayed recall test.

4. *The safe/unsafe classification needs justification as well: interactions are assessed but not required for a safe classification, recall of only one side effect is considered sufficient, and the outcomes refer to "dosage" although Table 1 reports frequency and administration instructions rather than an actual dose. Reporting component-level recall alongside the global classification would improve interpretation.*

   Response: We have clarified the safe/unsafe criteria as based on "the National Medication Standard" under the section Primary Safety Outcome: Medication safety (page 8). As interactions are not required for a safe classification, we have removed interactions from the coding plan.

We have also aligned the wording of medication usage items in both the procedure, fictional medications table, and outcomes to reflect: "frequency, timing, administration instruction, and side effects". (pages 5, 6, 8)

5. *A few aspects of teach-back, randomization, and outcome assessment also need clarification. Because actor-GPs correct incorrect responses and repeat correct responses during teach-back, delayed recall reflects modality plus standardized reinforcement rather than modality alone. Please clarify whether immediate recall is scored before feedback and how corrections, clarification requests, and protocol deviations will be documented.*

Response: We agree these are important points to address in the protocol. We now clarify the role of teachback and modality in delayed recall in the Outcome Measures section:

"Delayed memory accuracy reflects the effects of modality and standardized reinforcement during teach-back, rather than the effects of modality alone as captured in immediate memory accuracy. We will code teach-back content and participant questions during the consultation to allow for a closer examination of these factors in exploratory analyses." (page 9)

We also clarify when and how immediate recall and the simulated consultations are scored in a "data coding" section:

"Coders will also track participant questions during the consultation itself and code, for the item that the question is related to, the relevant question category: informational (seeking extra detail), clarification (seeking understanding), or advice (seeking related advice). These data may be used in exploratory analyses to better understand the role of clarification and information seeking in the delayed recall performance." (page 10)

6. *Participants are also randomized to one of two fictional medications that differ in instructions and side-effect content, so medication assignment should be included in the analysis.*

Response: We have added medication assignment as a covariate in the safety analysis plan (page 11). Note that we have not added medication assignment as part of sample size calculations, as the medications were piloted and designed to control for recall likelihood, such that we do not expect recall differences across medications.

7. *Allocation concealment is unclear because enrolment staff and actor-GPs appear to have access to allocation documents; please explain how foreknowledge of assignments is prevented. Finally, since the primary outcome is transcript-coded, please describe coder blinding, inter-rater agreement, and adjudication procedures.*

Response: We have endeavored to blind as much as possible in the study design. We address critical features of the study in detail below.

"Enrolment staff and coders will be blinded to consultation modality when enrolment/coding is taking place.

Blinding is impracticable for consultation staff, actor-GPs, and participants. Enrolment Consultation staff and actor-GPs will have access to an allocation document, which contains each participant's first name and the allocated modality and medication for each consultation. Participants are blinded to their consultation modality from enrolment until the consultation. They will not be informed of the two intervention conditions until after study completion." (page 7)

Regarding the transcript–coded outcome, we now note in the Data Coding section that "Coding disagreements will be resolved by the chief investigator C.P. as adjudicator. In the pilot study, the appointed coders reached inter-rater agreement of 100% after resolving disagreements and correcting errors." (page 11)

8. *The sample size and analysis plan are not fully aligned. The sample size calculation uses simple logistic regression with a one-tailed test, whereas the planned primary analysis uses mixed-effects logistic regression with actor-GP as a random intercept. Please justify this alignment, the assumed effect size, the one-tailed test, and the use of a random effect with only four actor-GPs.*

Response: We have aligned the sample size analysis and primary safety outcome analysis plan to both use simple logistic regression with a one-tailed test. As noted in point 3, the primary outcome that informed sample size is delayed recall accuracy.

We have now also included actor-GP as a random intercept in the secondary analysis plan for comparing safety across modalities as a robustness analysis, as GP clustering is not applicable to sample size calculation because 1) cluster size is variable (depending on availability) and 2) modality allocation is not fully randomized across GPs for this reason.

9. *Selecting covariates through univariate p values is generally not recommended for a randomized trial primary analysis; any adjusted covariates should be prespecified.*

Response: We have now specified that potential covariates include: "medication assignment, recall confidence, audio quality, video quality (only for video modality), age, and gender". (page 11)

10. *The missing-data plan also needs clarification, since excluding phase 2 non-completers may bias delayed-recall comparisons, and MICE alone does not address Missing Not at Random assumptions.*

Response: Thank you for identifying that simple exclusion of phase 2 non-completers may bias delayed-recall comparisons. In-line with standards often used in psychology research, we now note that:

"Participants who do not complete phase 2 will be excluded from analysis of delayed memory accuracy. If the number of phase 2 non-completers exceeds 10% of the total sample, we will assess potential bias introduced by differential attrition between conditions by comparing available sample baseline characteristics (age, end-of-consultation survey completion rate, experimenter-assessed technical difficulty of consultation) between completers and non-completers using chi-square tests and independent samples t-tests. We will report the proportion and characteristics of excluded cases. All other randomised participants will be included in data analysis below." (page 10)

11. *Several protocol reporting details remain incomplete. The patient-reported measures need clearer description, particularly the therapeutic alliance measure cited as a manuscript in preparation, which cannot be independently appraised.*

Response: We have added description of aims and items in the therapeutic alliance measure. (page 9)

12. *Please provide scoring, timing, missing-item handling, and available validity and reliability information for each measure; adapted GP-rated versions should be considered exploratory unless validated.*

Response: We have added scoring details to each measure under Primary Quality Outcomes. The timing of each measure is outlined in the Procedure section, while missing data handling is outlined in the Missing Data section. We have also changed the GP-rated measures to fall under exploratory analysis. (page 9)

13. *Please also clarify actor-GP training and fidelity monitoring, technical disruptions, and whether feedback after the first five consultations may alter delivery during data collection.*

Response: We have clarified actor-GP training in "Simulated Consultations" under Procedure, where "Actor-GP training aims to maximise GP authenticity while following a standardised delivery style to limit information exchanged to contents of the training manual: the consultation script and answers to common questions." (page 5).

We also clarify fidelity monitoring/feedback in "Data Monitoring" where "GP team members will review recordings of the first five consultations held by each actor-GP to provide feedback that aligns consultation delivery manner with the standardised procedure instructed during actor-GP training." (page 10).

We now capture participant experiences of technical disruptions in the phase 2 survey.

14. *Finally, please report recruitment status and study dates, clarify whether the trial was prospectively registered in a registry acceptable under journal policy, and strengthen the harms section by describing distress monitoring and debriefing, particularly given the inclusion of "intrusive suicidal thoughts" in the fictional medication information.*

Response: We have clarified recruitment status and study dates under “Study Timeline” in the Methods (page 7). Although this study is a non-clinical trial where prospective registration is suggested but not mandatory, we have clarified that the trial was prospectively registered under “Trial Registration and Data Statement” (page 2). We have also described distress monitoring strategies in more detail under Harms. (page 7)

15. *A few smaller points to consider as well. The title would benefit from indicating that the study involves simulated consultations and fictional medication information.*

    Response: Thank you for your suggestion. We have added these to the title. (page 1)

16. *The Discussion reports an age range of 40 to 65 years, whereas the eligibility criteria specify 18 to 44 years.*

    Response: Thank you for noticing this error which now has been corrected.

17. *Professor Bruce Christensen appears in the Author Statement but not in the author list, so authorship status should be clarified.*

    Response: We have updated the Authorship status of Professor Bruce Christensen.

18. *Please provide competing-interest disclosures for all investigators and state the funder's role.*

    Response: We have updated the competing-interest disclosure to involve all authors and stated the funder’s role under Funding Statement. (page 1)

19. *Terminology should be consistent for telephone/audio consultations and for de-identified/anonymous data.*

    Response: We have ensured all ‘audio consultations’ are termed ‘telephone consultations’.

20. *The Fakhrai reference also appears incomplete.*

    Response: We have corrected this reference.

VERSION 2 - REVIEW

**Reviewer**	**1**
**Name**	**Heidari-Soureshjani, Reza**
**Affiliation**	**Tehran University of Medical Sciences School of Nursing and Midwifery**
**Date**	**09-Aug-2026**
**COI**	

Thank you for the careful revision. Several issues from the first review have been addressed. However, a few important methodological concerns remain:

1. The primary outcome is still unclear. Delayed recall appears to drive the sample-size calculation, but immediate recall and several quality outcomes are also described as primary. Please identify one primary endpoint and time point, or clearly address multiplicity if multiple primary outcomes are retained.

2. The “safe/unsafe” classification needs stronger justification. A participant can be classified as safe after recalling only one side effect, even if a more serious side effect is missed. The cited medication-safety standard does not appear to validate this specific cutoff. Please provide a clinical or empirical basis for the threshold, or consider describing the outcome as adequate/inadequate medication-information recall.

3. The sample-size assumptions should be reconsidered. The 40% baseline estimate comes from a pilot outcome that differs from the composite outcome used in this trial. The assumed 65% versus 40% difference and the use of a one-sided test also require justification, and anticipated loss to the 48-hour follow-up should be considered.

4. The primary statistical model needs revision. Covariates are currently selected using univariate p<0.25, and some proposed covariates (e.g., recall confidence and audio/video quality) are measured after randomization and may themselves be affected by consultation modality. Primary adjustment should instead be prespecified and based mainly on appropriate baseline/design variables. The analysis should also account for the actor-GP stratification used in randomization.

Addressing these issues would substantially strengthen the protocol and the interpretability of the eventual trial findings.

Thank you for your great job.

VERSION 2 - AUTHOR RESPONSE

Thank you for your second review of our protocol paper entitled "Comparing the safety and quality of video versus telephone telehealth using simulated consultations on fictional medications for an Australian community sample: a randomised controlled trial protocol" (bmjopen-2026-121225). We are delighted to have the opportunity to revise and resubmit our protocol paper. We have carefully considered each point of feedback, and incorporated those changes along with our responses. We detail those edits below.

Comments to the Author:
Thank you for the careful revision. Several issues from the first review have been addressed. However, a few important methodological concerns remain:
1. The primary outcome is still unclear. Delayed recall appears to drive the sample-size calculation, but immediate recall and several quality outcomes are also described as primary. Please identify one primary endpoint and time point, or clearly address multiplicity if multiple primary outcomes are retained.

Thank you for identifying this issue. While immediate recall and several quality outcomes are all of interest, we agree that limiting the single primary outcome to delayed recall, which drove the sample size calculation, would increase clarity. We have reframed the other outcomes as secondary. (pages 8-9)

2. The "safe/unsafe" classification needs stronger justification. A participant can be classified as safe after recalling only one side effect, even if a more serious side effect is missed. The cited medication-safety standard does not appear to validate this specific cutoff. Please provide a clinical or empirical basis for the threshold, or consider describing the outcome as adequate/inadequate medication-information recall.

Thank you for suggesting classification terms that improve interpretation of the trial findings. We now describe the binary classification of consultations as adequate/inadequate medication-information recall on pages 8-9, and throughout the paper, noting that this term is used as a proxy for consultation safety.

3. The sample-size assumptions should be reconsidered. The 40% baseline estimate comes from a pilot

outcome that differs from the composite outcome used in this trial. The assumed 65% versus 40% difference and the use of a one-sided test also require justification, and anticipated loss to the 48-hour follow-up should be considered.

Our team has carefully reviewed sample-size calculations and we now provide more justification for each point in the paper.

The 65% vs 40% difference is powered to detect the sufficiently important difference (SID) which would justify the risks and potential harms associated with the use of video and the downgrading of telephone consultations, as assessed by policy makers, drawing on the arguments of Barrett et al (2013 and 2005). Significantly important differences are relevant for complex interventions as they take into consideration risk/benefit considerations. Page 7 now includes:

"The size of the projected difference used in calculations is derived from consideration of the estimated sufficiently important difference (SID). The SID is the smallest clinical benefit to a patient which would justify the associated costs, risks and harms of implementing the more beneficial method. In this case, we have estimated, in conversation with policy-makers, that a difference of 25% would be significant enough to warrant the costs and risks of further drivers of uptake of video and reduction in telephone consultation."

As there is no direct evidence to suggest a one-directional modality effect, we now use a two-tailed test to compare modalities, with a conventional power of 80%.

Finally, to address any loss over the 48-hour delay between study phases, we now state that "We will continue to recruit participants until valid data from participants who complete both phases reaches the desired sample size (N = 126; 63 participants per modality)" (page 7)

4. The primary statistical model needs revision. Covariates are currently selected using univariate p<0.25, and some proposed covariates (e.g., recall confidence and audio/video quality) are measured after randomization and may themselves be affected by consultation modality. Primary adjustment should instead be prespecified and based mainly on appropriate baseline/design variables. The analysis should also account for the actor-GP stratification used in randomization.

We agree that some proposed covariates may be affected by consultation modality. We have removed these variables (recall confidence and audio/video quality) from covariates and now assess the effect of modality on these variables in exploratory analysis on page 12: "We may also explore other outcomes between modalities, including but not limited to: recall confidence, audio quality, video quality (only for video modality), participant questions during the consultation, and actor-GP feedback during the consultation."

While participants are randomly allocated to modality and fictional medication, the allocation of participants to actor-GPs is not randomized—instead, actor-GP allocation depends on participant and actor-GP availability and timeline of recruitment signup. Thus, the role of actor-GP cannot be investigated as a fixed effect. Instead, we "examine the robustness of the primary safety analysis across actor-GPs," using "logistic regression models for safety outcome with modality (telephone vs. video) as the fixed effect and actor-GP as a random intercept to account for clustering of consultations within practitioners". (page 11)

Addressing these issues would substantially strengthen the protocol and the interpretability of the eventual trial findings.
Thank you for your great job.
