# Supplementary material for: Comparing the safety and quality of video versus telephone telehealth using simulated consultations on fictional medications for an Australian community sample: a randomised controlled trial protocol
Source: BMJ Open. 2026 Sep 30;16(9):e121225. doi: 10.1136/bmjopen-2026-121225 (PMC13629935; doi:10.1136/bmjopen-2026-121225)
Supplement: Supplementary file 2 [file bmjopen-16-9-s001.docx]

Data management plan

*A Data Management Plan created using DMP Tool*

Title**:** QuEST - How people experience and think about telehealth consultations

Creator**:** Megan Reid Hobbs

Affiliation**:** Australian National University (anu.edu.au)

Principal Investigator**:** Christine Phillips

Data Manager**:** Yangxueqing Jiang

Funder**:** National Health and Medical Research Council (nhmrc.gov.au)

Project abstract**:**

This research project aims to compare audio (telephone) versus video telehealth consultations to determine which consultation format leads to better patient outcomes when discussing high-risk medications. We will recruit a community sample from ACT Australia (N=130-150) who will attend simulated consultation sessions at ANU in either an audio or video format. During each consultation with a GP, participants will learn about medication usage, risks, and monitoring requirements. Immediately after each consultation, participants complete questionnaires measuring their consultation experience on scales measuring therapeutic alliance, perceived clinician empathy, behavioural intentions, and risk perceptions. Two to three days later, participants complete an online questionnaire in which they complete a structured recall of the medication information discussed. Outcome measures include patient understanding, memory retention, trust in healthcare providers, and overall consultation quality. We expect to identify which consultation format—audio or video—produces better patient outcomes, stronger therapeutic relationships, and safer medication management.

Start date**:** 07-01-2025

End date**:** 06-30-2027

Last modified**:** 07-09-2025

# Data Collection

- Personal information: first name, phone number, email address, gender, age of all participants (up to 150 participants), native English language status, country of birth and country of primary school education will all be collected via Qualtrics survey and verbally.
- End of consultation and delayed recall survey responses will be collected via Qualtrics.
  - End of consultation scales will include questions to measure therapeutic alliance, perceived clinician empathy, behavioural intentions, and risk perceptions.
  - Delayed recall will include structured recall questions on medication dosage, timing, duration and mode of administration, adverse effects, interactions, and monitoring.
- Simulated consultations will be audio/video recorded and transcribed verbatim via Microsoft Teams.
- Questionnaire data will be downloaded via Qualtrics in the form of excel files which contain all responses to the three questionnaires (demographic information, end of consultation and delayed recall).
  - These will be combined using participant emails, which will then be replaced with unique identifiers (24-character alphanumeric identifier).
- Recordings and transcriptions will be downloaded from Microsoft Teams at the conclusion of each session and named per the participants unique identifier (de-identified).
- A SharePoint structure has been developed which allows for easy and secure storage of data and follows common conventions used previously by the research team.
- Version control is managed within SharePoint, so each file will be edited, and previous revisions can be accessed, but no duplicated files will be created.
- The research administrator will provide support to ensure quality assurance and that consistency and quality of data collection will be controlled and documented.
- Naming conventions have been developed and shared as a team resource.

# Documentation and Metadata

- All data are stored in excel, with guidance on how to interpret result outputs on the first tab of each spreadsheet.
- Pre-registration: how we plan to analyse the data to assist reviewers in understanding our analytical approach and intent.
- Analytical tools to assist interpretation of the data, including "safe/unsafe" side effect measures and scoring metrics for recall, as well as all other variables.
- All documents will be saved to secure ANU SharePoint sites created for the QuEST project, with access restricted to the research team.
- Naming convention tool to assist in quality storage of data.

# Ethics and Legal Compliance

- Ethics approval for this research project have been sought from ANU HREC. Ethical issues will be managed in line with this application (approval number tbc).
- Data are owned by ANU, and ANU is committed to compliance with the Copyright Act 1968 and upholding the rights of creators and users of copyright material.

# Storage and Backup

- All data will be stored securely on SharePoint (OneDrive), per ANU policies.
- Data access will be secured so only researchers who have been given permission within the system can access the files. This access will be limited to those undertaking analysis, etc.

# Selection and Preservation

- Personal information including name, email and phone number will be destroyed following conclusion of data collection and prior to data analysis.
- As per research ethics and legislative guidelines, all other data (questionnaires and recording/transcriptions) will be kept for a minimum period of five years after the completion of the study. Beyond the initial storage period, the anonymous data will remain archived on publicly accessible data repositories, so other researchers have access as per journal requirements.
- No costs are required to support the minimum five-year storage of relevant data.
- Data will be stored in secure SharePoint (OneDrive) files.

# Data Sharing

- The data collected in this project may be utilised for future research activities. Data may be shared with other researchers who are part of the broader study team within the NHMRC Grant. This sharing will support the writing of grant reports, future discussions, and provide critical pilot data and foundational evidence for other larger research programs and grant applications in future public health or telehealth research.
- Once the research paper(s) are published, some journals may require that data be made publicly available. Only de-identified, anonymous research data will be made available on open science websites to ensure compliance with journal requirements and Open Access policies such as those of the NHMRC and the Australian Research Council.
- During the consent process, participants will grant consent for the data to be used to produce peer-reviewed published articles and may be used in conference presentations or as data that form part of future research proposals.
- Any use beyond this is restricted.

# Responsibilities and Resources

- Yangxueqing Jiang (data manager), Christine Phillips (primary investigator), and Megan Reid Hobbs (administrator).
- Any expertise required is already held within the team, and opportunities for further upskilling will be utilised if need is identified.
- Required software is already provided via ANU subscriptions and access.

# Planned Research Outputs

| **Title** | **Type** | **Anticipated release date** | **Initial access level** | **License** | **Metadata standard(s)** | **May contain sensitive data?** | **May contain Personal ID Info?** |
| --- | --- | --- | --- | --- | --- | --- | --- |
| **Other academic journal publications** | Data paper | Unspecified | Open | None specified | None specified | No | No |
| **End of consultation** | Dataset | Unspecified | Restricted | None specified | None specified | No | Yes |
| **Delayed recall** | Dataset | Unspecified | Restricted | None specified | None specified | No | Yes |
| **Recordings of consultations** | Audiovisual | Unspecified | Restricted | None specified | None specified | No | Yes |
| **Transcriptions of consultations** | Text | Unspecified | Restricted | None specified | None specified | No | No |
| **NHMRC Tools and Resources** | Interactive resource | Unspecified | Open | None specified | None specified | No | No |
| **Protocol publication** | Text | Unspecified | Open | None specified | None specified | No | No |
| **Participant Demographics** | Dataset | Unspecified | Restricted | None specified | None specified | Yes | Yes |
| **Scheduling document** | Text | Unspecified | Restricted | None specified | None specified | Yes | Yes |
